# Update: Ebola Virus Disease Epidemic — West Africa, November 2014

**Published:** 2014-11-21

**Authors:** Incident Management System Ebola Epidemiology Team, CDC; Guinea Interministerial Committee for Response Against the Ebola Virus and the World Health Organization; CDC Guinea Response Team; Liberia Ministry of Health and Social Welfare; CDC Liberia Response Team; Sierra Leone Ministry of Health and Sanitation; CDC Sierra Leone Response Team; Viral Special Pathogens Branch, National Center for Emerging and Zoonotic Infectious Diseasesm CDC

CDC is assisting ministries of health and working with other organizations to end the ongoing epidemic of Ebola virus disease (Ebola) in West Africa (1). The updated data in this report were compiled from situation reports from the Guinea Interministerial Committee for Response Against the Ebola Virus and the World Health Organization, the Liberia Ministry of Health and Social Welfare, and the Sierra Leone Ministry of Health and Sanitation. Total case counts include all suspected, probable, and confirmed cases, which are defined similarly by each country (2). These data reflect reported cases, which make up an unknown proportion of all cases, and reporting delays that vary from country to country.

According to the latest World Health Organization update on November 14, 2014 (3), a total of 14,383 Ebola cases have been reported as of November 11 from three West African countries (Guinea, Liberia, and Sierra Leone) where transmission is widespread and intense. The highest reported case counts were from Liberia (6,878 cases) and Sierra Leone (5,586), followed by Guinea (1,919). Peaks in the number of new cases occurred in Liberia (509 cases), Sierra Leone (540 cases), and Guinea (292 cases) at epidemiologic weeks 38 (September 14–20), 44 (October 26–November 1), and 41 (October 5–11), respectively (Figures 1 and 2). A total of 5,438 deaths have been reported. Investigation of localized transmission in two locations in Mali (Kourémalé and Bamako) is currently underway (4). Transmission was interrupted successfully in Nigeria (October 19) and prevented in Senegal (October 17) (3).

The 2,705 new Ebola cases reported during October 19–November 8 were more widely distributed geographically among districts in Guinea and Liberia compared with the 2,809 new cases reported during September 28–October 18 (5). During both periods, counts of Ebola cases reported were highest in the area around Monrovia, Liberia; the Western and northwest districts of Sierra Leone, particularly Bombali and Port Loko; and the prefectures of Kérouané, Macenta, and Nzérékoré, Guinea (Figure 3).

As of November 8, the highest cumulative incidence rates (>100 cases per 100,000 population) were reported by two prefectures in Guinea (Guéckédou and Macenta), four counties in Liberia (Bomi, Lofa, and particularly Margibi and Montserrado), and five districts in Sierra Leone (Bombali, Kailahun, Kenema, Port Loko, and Western Area) (Figure 4). Evidence of decreasing incidence in Lofa and Montserrado, Liberia, is described elsewhere (6–8).

The latest updates on the 2014 Ebola epidemic in West Africa, including case counts, are available at http://www.cdc.gov/vhf/ebola/outbreaks/guinea/index.html. The most up-to-date infection control and clinical guidelines on the 2014 Ebola epidemic in West Africa are available at http://www.cdc.gov/vhf/ebola/hcp/index.html.

## Figures and Tables

**FIGURE 1:**
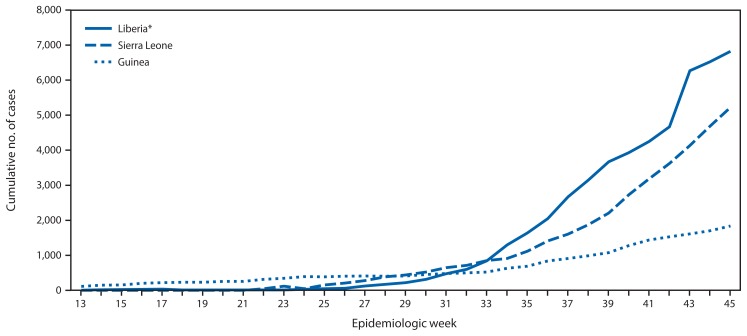
Cumulative number of Ebola virus disease cases reported, by epidemiologic week — three countries, West Africa, March 29–November 8, 2014 * A change in reporting source data at week 43 resulted in an adjustment of cumulative cases in Liberia.

**FIGURE 2:**
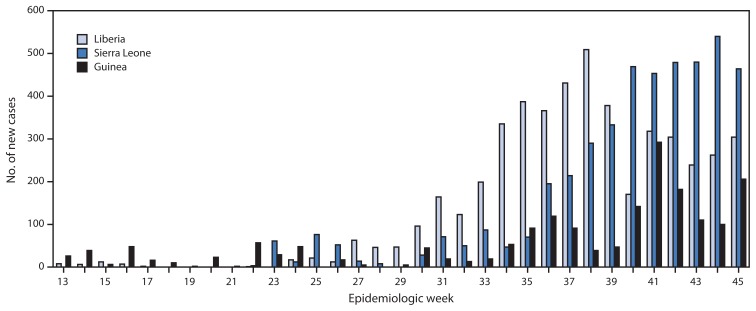
Number of new Ebola virus disease cases reported, by epidemiologic week — three countries, West Africa, March 29–November 8, 2014

**FIGURE 3:**
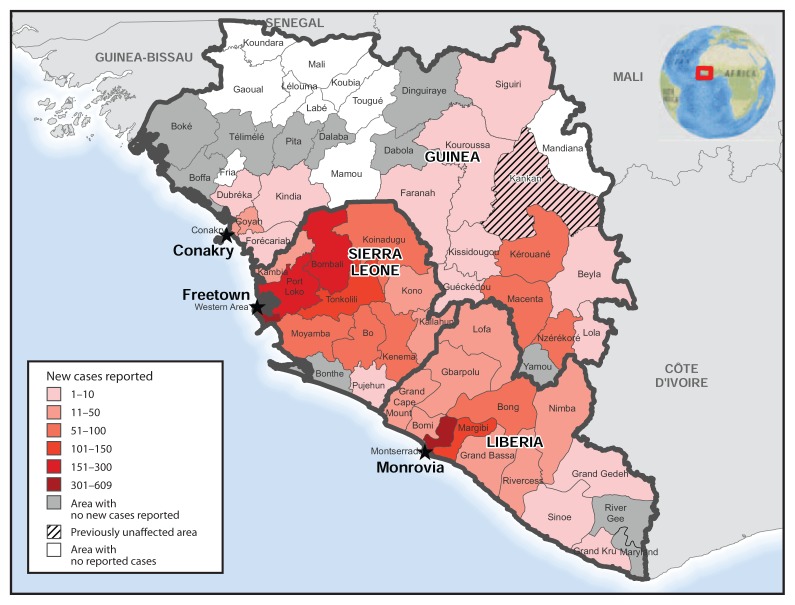
Number of new cases of Ebola virus disease reported — Guinea, Liberia, and Sierra Leone, October 19–November 8, 2014

**FIGURE 4:**
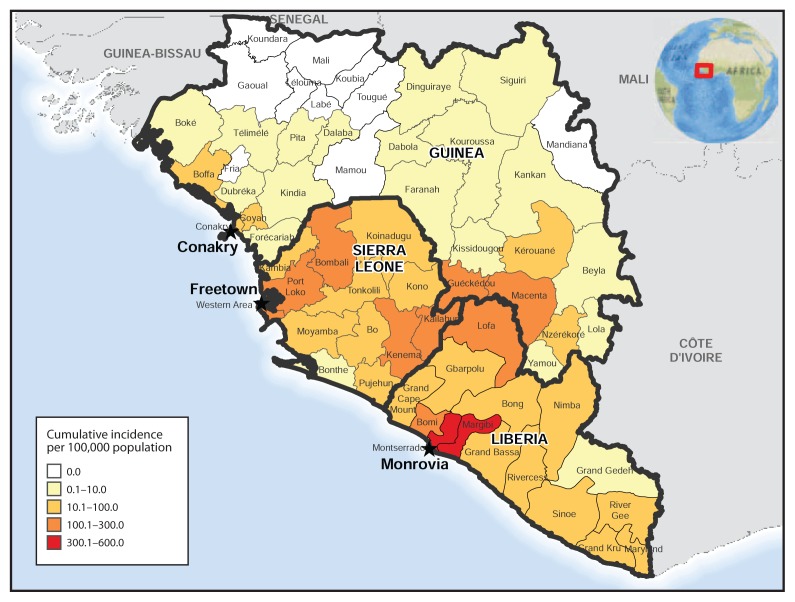
Cumulative incidence of Ebola virus disease — Guinea, Liberia, and Sierra Leone, November 8, 2014

## References

[1] Dixon MG, Schafer IJ (2014). Ebola viral disease outbreak—West Africa, 2014. MMWR Morb Mortal Wkly Rep.

[2] World Health Organization Case definition recommendations for Ebola or Marburg virus diseases.

[3] World Health Organization (2014). Ebola response roadmap situation report.

[4] CDC (2014). Ebola Outbreak in West Africa—case counts.

[5] Incident Management System Ebola Epidemiology Team, CDC; Ministries of Health of Guinea, Sierra Leone, Liberia, Nigeria, and Senegal; Viral Special Pathogens Branch, National Center for Emerging and Zoonotic Infectious Diseases, CDC (2014). Ebola virus disease outbreak—West Africa, October 2014. MMWR Morb Mortal Wkly Rep.

[6] Sharma A, Heijenberg N, Peter C (2014). Evidence for a decrease in transmission of Ebola virus—Lofa County, Liberia, June 8–November 1, 2014. MMWR Morb Mortal Wkly Rep.

[7] Nyenswah TG, Westercamp M, Ashraf Kamali A (2014). Evidence for declining numbers of Ebola cases—Montserrado County, Liberia, June–October 2014. MMWR Morb Mortal Wkly Rep.

[8] Nyenswah T, Fahnbulleh M, Massaquoi M (2014). Ebola epidemic—Liberia, March–October 2014. MMWR Morb Mortal Wkly Rep.

